# Disparity in physician-patient communication by ethnicity: evidence from Bangladesh

**DOI:** 10.1186/s12939-021-01405-6

**Published:** 2021-02-24

**Authors:** Muhammad Zakaria, Rezaul Karim, Murshida Rahman, Feng Cheng, Junfang Xu

**Affiliations:** 1 Department of Communication and Journalism, University of Chittagong, Chittagong, 4331 Bangladesh; 2 Department of English, Hamdard University Bangladesh, Munshiganj, 1510 Bangladesh; 3 Vanke School of Public Health, Tsinghua University, Beijing, 100084 China; 4 Center for Health Policy Studies, School of Public Health, Zhejiang University School of Medicine, Hangzhou, 310058 China

**Keywords:** Physician-patient communication behavior, Ethnic disparity, Bengali, Ethnic minority, Chittagong Hill tracts, Bangladesh

## Abstract

**Background:**

Physician-patient communication behavior (PPCB) is the primary process by which medical decision-making occurs and health outcome depends. Physician-patient communication differences may partly from the ethnic disparities. To examine this problem, this study aims to explore whether physician-patient communication differs by ethnicity during primary care medical consultations.

**Methods:**

The study was conducted among the Bengali and ethnic minority patients (*N* = 850) who visited a physician for medical consultations. Data were collected using a structured post-consultation questionnaire. T-test was conducted to compare the communication between the Bengali and ethnic minority patients. Multiple linear regression analyses were performed to identify the factors associated with favorable communication behavior from the physicians.

**Results:**

Bengali patients received more supportive communication behaviors from the Bengali doctors than that of ethnic minority patients including physicians’ cheerful greetings, encouraging patients to express health problems and asking questions, listening carefully, responding to questions and concerns, explaining to patients about medical examination procedures, medication, probable side effects, discussing treatment options, involved the patients in decisions, and spending adequate time. Results of linear regression showed that respondents’ level of education, internet use, knowledge about the health issue, having a pre-organized plan about the content of medical consultation, information seeking about the health problem, visiting female doctors, and a quiet ambience of the doctor’s room are significantly associated with a better PPCB score for the Bengali patients. In contrast, age, being the resident of an urban area, perception of affecting a minor health problem, having a pre-organized plan about the content of medical consultation, patients’ involvement in physicians’ decision-making about the treatment, and talking time resulted in better physician-patient communication for the ethnic minority patients.

**Conclusion:**

This study suggests that reducing disparity in the socio-economic status of the ethnic minority groups through development programs and educating healthcare providers on how to use patient-centered communication skills to engage with their patients is one solution to improve equity in the delivery of healthcare and ensure than patients are receiving high-quality treatment, no matter their race or ethnicity.

## Introduction

Physician-patient communication behavior (PPCB) is the primary process for health care service utilization. Research shows that successful PPCB can contribute to enhanced patient satisfaction and adherence to treatment and health outcomes, decreased costs of medical malpractice, and increased work satisfaction for physicians [1–4]. In addition, positive doctor-patient interactions could promote the sharing and obtaining of knowledge and overcome any differences of opinion during a physician’s visit. However, creating a productive PPCB relationship is difficult when the doctor and the patient come from different racial or ethnic groups [1] because of various factors (i.e., language barriers and paradoxical attitudes about disease and illness). There is a growing body of empirical evidence showing that ethnic minority patients are received less patient-centered behavior from the discordant ethnic physicians and other health professionals, as cultural disparities between patients and physicians create trust barrier between the two parties, posing potential obstacles to the adequate provision of health services [3, 5–12]. Thus, failure to solve these problems may result in poor health outcomes for patients.

Racial or ethnic disparities in healthcare are defined as “differences in the quality of healthcare which are not due to access-related factors or clinic needs, preferences, and appropriateness of intervention” [13]. Ethnicity and culture influence the health outcomes through communicative actions of providers and patients in at least three ways [11]. Firstly, people of different ethnic backgrounds often speak different languages or dialects, even when the same language is generally spoken; there may still be cultural-specific uses of it, which contributes to communication related problems between physicians and patients. Secondly, preferred communication styles can vary across various cultural groups, particularly in regards to assertiveness and expressiveness. Finally, people of different ethnic backgrounds may have different methods of explanation regarding personal health and diseases and these, in turn, can affect the nature of medical consultations. These inequalities are exacerbated by prejudice about racism along with healthcare variables at the systemic level, such as language barriers, time constraints and regional disparities in quality of health treatment.

Previous studies have shown that cultural awareness, personal style, and language gaps often influence the outcome of clinical visits. For example, among a sample of general practice patients in the Netherlands, Harmsen et al. [14] examined the impact of cultural differences on mutual understanding and compliance, which found that interactions in consultations between general physicians and ethnic minority individuals were less effective than consultations with Dutch individuals. Some research [15, 16] indicated that contact with patients of various cultural backgrounds can also be troublesome, as doctors do not want to discuss cross-cultural issues. This could be for several reasons, including fear of sounding racist or prejudiced, a sense of inadequacy, a lack of cultural awareness, a fear of confusion or being rejected if suggestions are not culturally appropriate, and even ambiguity about whether or not the patient is an immigrant.

In Bangladesh, more than 45 ethnic minorities existed in the country prior to independence in 1971 [17]. Some cross-cultural or cross-ethnic studies in Bangladesh show that due to their low socio-economic status, minority groups often struggle to manage their livelihoods, which leads to deprivation of basic amenities, discrimination and health problems [18, 19]. Although these minority communities have their own language, they are unable to use their language in every aspect of life due to the dominance or hegemony of Bengali language for its acceptance and inadequacy of their language. For example, people from ethnic minority groups may encounter language problems when admitted to a hospital or when visiting physicians. Even though the minority communities are gradually shifting to the Bengali language for survival in society, they may speak Bengali with a non-native accent which may lead to experiencing communicative difficulties and other ethnicity related problems when accessing health services.

Incorporating ethnic contexts into health communication research in health care is an important part of promoting health equity between the majority Bengali group and ethnic minority groups in Bangladesh [20, 21]. Tufnell et al. [22] observed that more than a third Bangladeshi population could not read or write in Bengali. This issue was more noteworthy among the older members in Bangladesh. It not only impedes communications with physicians but also means that these patients are unlikely to benefit from additional written medical materials. Moreover, Paternotte et al. [23] showed that language differences literally caused miscommunication in which it is not possible for the physician to achieve shared decision-making [23]. However, most of the related studies were conducted in western countries under primary care settings, including the USA and the UK [24], while very little study has been carried out in developing countries especially Bangladesh. The little existing studies in Bangladesh only focused on patients’ satisfaction [25]; they did not examine the physician-patient relationship, communication between two parties, and its’ link with cultural disparity. Under this background, this study aims to explore whether physician-patient communication differs by ethnicity in primary care medical consultations and then examine the factors influencing better communication behavior in primary care medical consultations in Bangladesh. The importance of this study lies in filling the research gap in this regard. Findings may also inform the public health practitioners about the existing communication patterns between physicians and patients by ethnic discordance that might serve as barriers of good compliance.

## Methods

### Study design

A hospital based cross-sectional survey was used to investigate the patients. This study was conducted in three district hospitals and three Upazila (sub-district) hospitals in the Chittagong Hill Tracts area (CHT). All the patients including Bengali and ethnic minority patients who visited physicians for primary care medical consultations in the study settings from August 19 to September 30, 2019 were incorporated to our study population.

Bangladesh is divided into 64 districts and the districts are further subdivided into 493 sub-districts or Upazila [26]. In each district, there is a district hospital which is responsible for delivering secondary and primary-care services to the whole district, whereas, at the Upazila level, there is an Upazila hospital or Upazila Health Complex (UHC) that provides all public-health programs, particularly primary healthcare services for the Upazila [27].

### Sample size and sampling procedures

For this hospital based cross-sectional study, purposive sampling method was used to select the study areas, while study participants were selected using a systematic sampling method. Patients from three district hospitals and three Upazilas in three districts (*Rangamati* districts, *Bandarban* district, and *Khagrachori* district) under CHTs area were taken as participants. Finally, a total of 850 respondents were investigated and incorporated in the analysis. The proportion of participants [400 (47.1%) were Bengali while 450 (52.9%) were from three ethnic minority groups] in our study was in accordance with the overal population proportions of ethnicity in Bangladesh.

### Data collection

Data were collected using a structured post-consultation questionnaire composed in the Bangla language. For pre-testing the questionnaire, 20 patients from the district hospital and 20 from an UHC were randomly selected. The questionnaire included the following four parts, a) patients’ demographic and socio-economic characteristics; b) patients’ cognitive, affective influences; c) physicians’ predisposing and organizational context; d) physician-patient communication behavior (PPCB). PPCB was assessed using a scale that consists of 19 items developed by Wachira et al. [28], which consists of physicians’ cheerful greetings, encouraging patients to explain their health problems and ask questions, giving information according to patients’ needs, responding to questions and concerns, explaining to patients about medication, involving patients in decisions, and spending adequate time. To ensure the content was applicable to the context of Bangladesh, the scale was translated into the local language (i.e., Bangla) for appropriateness and convenience. Thereafter, the Bangla version was translated back to English to ensure consistency and accuracy of meaning. The translation of the questionnaire was performed by language experts in both cases. The appropriateness of the items of the scale was supported by adequate internal reliability (α = .76). Five-point Likert scale was used to measure each item related to physician-patient communication behavior as strongly disagree =1, disagree =2, neither agree nor disagree = 3, agree = 4, strongly agree = 5.

The survey was guided and administered by six facilitators who were employed based on prior experience regarding data collection and eloquence in the local accent of the Bangla language for both Bengali and ethnic minority groups. Respondents were asked to respond to the questions at the end of their medical consultation. The informed written consent for participating in the study was obtained from each of the participants. Data collectors checked the filled questionnaire and asked the study participants to complete the questions if there was any missing information. Accordingly, there were no missing values in the study.

### Statistical analysis

Cross-tabulation with chi-square (*χ2*) analysis was used to examine the relationship between socio-demographic and other descriptive characteristics by respondents’ ethnicity. Then, a two-tailed t-test was conducted to see the statistically significant differences between the Bengali and ethnic patients related to PPCB. Multiple linear regression analyses were also used to examine the factors influencing PPCB for the Bengali and ethnic minority groups. The score of 19 items regarding physician-patient communication behavior (PPCB) were computed and the overall PPCB score was the outcome variable. The distribution of the score of dependent variable was approximate normal distribution. Thus, the variables with a *p* < .05 in t-test and ANOVA were included in the linear regression models to determine the predictors of better PCCB. ANOVA values for better PPCB for the Bengali (*F* = 8.66, *p* < .001), and that for ethnic minority (*F* = 15.38, *p* < .001) showed that our multiple linear regression model performed well and would be a good predictor of the main outcome variable. Variables with *p* < 0.05 were considered as statistically significant.

## Results

### Socio-demographic characteristics of participants by ethnicity

Table 1 shows the socio-demographic characteristics of the study participants by their ethnic identity. Of them, 228 (26.8%) had no formal education, while only 92 (10.8%) had above 12 education years. Among the participants, 250 (29.4%) were > 20–30 years, whereas 168 (19.8) were > 40 years old. Besides, 499 (58.7%) were male and 351 (41.3%) were female. For working industry, among the Bengali participants, 22% were involved in agriculture, 14.3% were in service, and 15.5% in business, while for the ethnic minority respondents, it was 29.3% (agriculture), 8.4% (service) and 9.6% (business) respectively. Among the Bengali participants, 19% had an income of BDT > 30,000 (good income), while only 4.7% of ethnic minority participants had this amount of income.

**Table 1:** Respondents’ socio-economic characteristics by ethnicity (*N* = 850)

Variable (***N*** = 850)	***n***	Bengali (%)	Ethnic Minority (%)	χ2	***p***
Gender				1.64	.200
Female	351	156 (39.0)	195 (43.3)		
Male	499	244 (61.0)	255 (56.7)		
Age (years)				3.27	.352
Up to 20	218	106 (26.5)	112 (24.9)		
> 20–30	250	114 (28.5)	136 (30.2)		
> 30–40	214	109 (27.3)	105 (23.3)		
> 40	168	71 (17.8)	97 (21.6)		
**Education**				8.04	.154
No education	228	110 (27.5)	118 (26.2)		
Up to class 5	113	53 (13.3)	60 (13.3)		
class > 5–8	131	69 (17.3)	62 (13.8)		
class > 8–10	163	70 (17.5)	93 (20.7)		
Class > 10–12	123	48 (12.0)	75 (16.7)		
Class > 12	92	50 (12.5)	42 (9.3)		
**Occupation**				18.52	.001
No job	170	81 (20.3)	89 (19.8)		
Agriculture	220	88 (22.0)	132 (29.3)		
Student	260	112 (28.0)	148 (32.9)		
Service	95	57 (14.3)	38 (8.4)		
Business	105	62 (15.5)	43 (9.6)		
**Monthly household income (BDT)**				85.33	<.001
Up to 5000	165	35 (8.8)	130 (28.9)		
> 5000–10,000	228	110 (27.5)	118 (26.2)		
> 10,001–20,000	255	121 (30.3)	134 (29.8)		
> 20,001–30,000	105	58 (14.5)	47 (10.4)		
> 30,000	97	76 (19.0)	21 (4.7)		
**Internet use**				0.05	.814
Yes	401	147 (46.8)	214 (47.6)		
No	449	213 (53.3)	236 (52.4)		

Columns against the categories of characteristics of each ethnic group sum to 100%. Chi-square (*χ2*) test was performed to depict the difference. *BDT* Bangladeshi Taka

### Patient and physician characteristics by ethnicity

Physicians’ predisposing, patients’ cognitive, and organizational characteristics according to the ethnic identity is reported in Table 2. The Bengali participants (32.8%) were more likely to be returning patients than their ethnic minority counterparts (25.3%). In regards to the perception of health-related knowledge, the Bengali patients were more likely to have a good level of knowledge (30.8%) compared to ethnic minority patients (18.4%). In the case of the perception about the ambiance of the physicians’ room, Bengali patients (17.8%) were more likely to mention it as ‘quiet’ relative to their ethnic minority counterparts (9.3%). Physicians had a higher likelihood to talk with the Bengali patients in comparison with ethnic minority patients. Moreover, ethnic minority patients were less likely to participate in medical consultations in contrast to Bengali patients (*p* < .01).

**Table 2:** Cognitive, organizational and other predisposing characteristics by the ethnicity

Variable (***N*** = 850)	***n***	Bengali (%)	Ethnic Minority (%)	χ2	***p***
**Patient Type**				5.68	.017
Returning	245	131 (32.8)	114 (25.3)		
New	605	269 (67.3)	336 (74.7)		
**Knowledge of the problem affected**				27.34	<.001
Poor	625	263 (65.8)	362 (80.4)		
Good	225	137 (34.3)	88 (19.5)		
**Perception of the severity of the problem**				4.87	.301
Slight	335	177 (44.3)	158 (37.3)		
Moderate	407	183 (45.8)	224 (49.8)		
Extreme	98	40 (10.0)	58 (12.9)		
**Waiting time (minutes)**				1.41	.495
> 0–20	293	132 (33.0)	161 (35.8)		
> 20–40	224	103 (25.8)	121 (26.9)		
> 40	333	165 (41.3)	168 (37.3)		
**Gender of the doctor**				4.52	.034
Female	228	121 (30.3)	107 (23.8)		
Male	622	279 (69.8)	343 (76.2)		
**The ambiance of the doctor’s room**				16.99	<.001
Noisy	459	218 (54.5)	241 (53.6)		
Average	278	111 (27.8)	167 (37.1)		
Quiet	113	71 (17.8)	42 (9.3)		
**Talking time (minutes)**				54.11	<.001
Up to 5	219	58 (14.5)	161 (35.8)		
> 5–10	197	180 (45.0)	17 (38.9)		
> 10	276	162 (40.5)	114 (25.3)		
**Patients’ information seeking about the health problem**				21.87	<.001
Yes	233	140 (35.0)	93 (20.7)		
No	617	260 (65.0)	357 (79.3)		
**Patients’ involvement in decision-making of the treatment**				6.96	.008
Yes	554	279 (69.8)	275 (61.1)		
No	296	121 (30.2)	175 (38.9)		

Columns against the categories of characteristics of each ethnic group sum to 100%. Chi-square (*χ2*) test was performed to depict the difference

### Difference between the Bengali and ethnic minority patients regarding physicians’ communication behavior

Table 3 demonstrates that the Bengali patients reported better physician communication behavior than the ethnic minority patients did for 18 out of the 19 items for PPCB (*p* ≤ .001), which included: physicians’ greeting the patients, encouraging to express the health problems, listening carefully to the patients, understanding the patients, explaining the physical examination, explaining the lab tests needed, discussing treatment options with the patients, giving adequate information, and checking that the treatment plan(s) were acceptable to the patients, among others. However, there was no significant difference by ethnicity in the mean score of the item whether physicians asked patients the reason(s) for coming (*t* = .04, *p* = .967).

**Table 3:** Mean score with standard deviation and independents samples t-test of different items relating to physician-patient communication behavior (PPCB) by ethnicity

Items	Bengali	Ethnic Minority	***t***	***p***
Your doctor greeted you in a way that made you feel comfortable	3.53 (±1.16)	3.27 (±1.09)	3.44	.001
Discussed your reason(s) for coming	4.04 (±).74	4.04 (±.80)	.04	.967
Encouraged you to express your thoughts concerning health problems	3.99 (±.76)	3.48 (±1.01)	8.41	<.001
Listened carefully to you	4.11 (±.77)	3.84 (±.87)	4.90	<.001
Understood what you had to say	3.93 (±.94)	3.62 (±1.04)	4.56	<.001
If a physical examination was required, the doctor fully explained	3.19 (±1.23)	2.47 (±1.24)	8.43	<.001
Explained the lab tests needed (e.g., blood, X-rays, ultrasound, etc.)	3.22 (±1.15)	2.45 (±1.26)	9.31	<.001
Discussed treatment options with you	3.73 (±1.00)	3.08 (±1.23)	8.54	<.001
Gave you as much information as you wanted	3.72 (±.92)	3.20 (±1.01)	7.77	<.001
Checked to see if the treatment plan(s) was acceptable to you	2.70 (±1.36)	2.14 (±1.32)	6.19	<.001
Explained medications, if any, including possible side effects	2.64 (±1.30)	2.19 (±1.25)	5.17	<.001
Encouraged you to ask questions	3.81 (±.86)	3.02 (±1.13)	11.57	<.001
Responded to your questions and concerns	3.73 (±1.01)	2.94 (±1.26)	10.14	<.001
Showed concern about you as a person	3.97 (±.82)	3.76 (±.94)	3.61	<.001
Involved you in decisions about your health as much as you wanted	3.56 (±.99)	3.17 (±1.13)	5.29	<.001
Discussed next steps including any follow-up plans	3.64 (±1.10)	2.83 (±1.35)	9.56	<.001
Checked to be sure you understood	3.63 (±1.09)	2.83 (±1.27)	9.84	<.001
Spent the right amount of time with you	3.98 (±.76)	3.43 (±.95)	9.34	<.001
Overall, you were satisfied with your visit to the doctor today	3.95 (±.83)	3.60 (±1.03)	5.40	<.001

### Factors associated with PPCB by the ethnicity

For Bengali patients, as Table 4 illustrates, respondents’ level of education (β = .14, *p* = .042), internet use (β = .16, *p* = .016), good knowledge about the health issue (β = .16, *p* < .001), having a pre-organized plan about the content of medical consultation (β = .12, *p* = .012), patients’ information seeking about the health problem (β = .17, *p* < .001), female physicians (β = .10, *p* = .035), and a quiet ambience of the physician’s room (β = .11, *p* = .029) are significantly associated with better PPCB. In contrast, for the ethnic minority patients, respondents’ age (β = .20, *p* < .001), being the resident of urban area (β = .09, *p* = .034), perception of affecting a minor health problem (β = .10, *p* = .016), having a pre-organized plan about the content of medical consultation (β = .17, *p* < .001), patients’ involvement in physicians’ decision-making about the treatment (β = .19, *p* < .001), and talking time (β = .35, *p* < .001) are significantly associated with better PPCB.

**Table 4:** Multiple linear regression analysis showing factors associated with physician-patient communication by the ethnicity

Variables	Bengali					Ethnic Minority				
	B	SE	β	***t***	***p***	B	SE	β	***t***	***p***
Constant	48.36	2.93		16.49	<.001	27.811	3.73		7.45	<.001
Education of the respondents (Continuous variable)	.158	.08	.14	2.00	.042	.180	.10	.11	1.76	.079
Occupation of the respondents (agriculture/no job vs. business/service/student)	.181	.80	.01	.23	.820	.963	1.10	.06	.88	.378
Age of the respondents (Continuous variable)	.048	.03	.10	1.65	.101	.120	.03	.20	3.61	<.001
Area of Residence (rural vs. urban/sub-urban)	.022	.63	.00	.03	.972	1.670	.79	.09	2.13	.034
Marital status of the respondents (married vs. single/widow/divorced)	1.164	.81	.09	1.43	.153	1.571	1.11	.09	1.42	.156
Respondents’ Internet use (no vs. yes)	1.937	.80	.16	2.42	.016	.970	.94	.06	1.03	.303
Perception of the problem’s severity (severe vs. minor)	−.285	.57	−.02	−.50	.616	1.724	.71	.10	2.42	.016
Knowledge of health problem (poor vs. good)	1.999	.61	.16	3.30	.001	−.708	.88	.03	−.81	.421
Plan about consultation before visit (no vs. yes)	1.498	.59	.12	2.53	.012	2.815	.71	.17	3.95	<.001
Patients’ information seeking (no vs. yes)	2.136	.58	.17	3.68	<.001	.303	.84	.01	.36	.718
Involvement in decision-making (no vs. yes)	.642	.61	.05	1.05	.292	3.231	.70	.19	4.60	<.001
Gender of the doctors (male vs. female)	1.322	.63	.10	2.11	.035	.228	.80	.01	.28	.777
Patient Status (new ns. returning)	.301	.60	.02	.50	.616	.477	.78	.02	.61	.539
Talking time (Continuous variable)	.056	.04	.06	1.37	.171	.530	.07	.35	8.08	<.001
Ambience of the doctor’s room (noisy vs. quiet)	1.762	.81	.11	2.19	.029	1.944	1.15	.07	1.69	.091
	*R*^*2*^ = .25		*F* = 8.66		<.001	*R*^2^ = .35		*F* = 15.38		<.001

## Discussion

Our study found that concordant ethnicity between physician and patient was a key contributor for better patient-centered communication behavior, which illustrated the ethnic minority patients were less likely to receive better communication behavior from the discordant Bengali physicians. For example, the Bengali patients were more likely to receive friendly greetings from the physician that made them more comfortable with the medical consultations than that of the ethnic minority patients. Physician-patient communication begins through the form of a greeting. If not received warmly by the physician, the ethnic minority patient may feel discomfort, which likely will affect the entire consultation and lead them to communicate be less actively with the physician.

In terms of the affectionate behaviors, for example, encouraging patients to express their thoughts concerning health problems, listening carefully to and understanding what the patient says, our study observed that ethnic discordant physicians showed less concern towards ethnic minority patients. Similarly, we found that ethnic discrimination from Bengali physicians to the ethnic minority patients exists when it comes to discussing treatment options with the patients and providing adequate information. However, patient participation is an important element for effective physician-patient communication. Yet, patient satisfaction depends on the response behavior of the physician. We found a high inconsistency by ethnic discordance regarding encouraging the patients to ask questions and replying to the concerns. The general finding that Bengali physicians are less favorable towards the ethnic minority patients might be caused by the difference of language and culture, which is worthy of a future qualitative study [11, 12, 28]. This result was consistent with previous research conducted in Texas, USA that showed that physicians used less supportive talk or less patient-centered with non-white patients [11].

It is also reported that there was a vast discordance relating to the physicians’ discussion on next steps, including any follow-up plans and checking to be sure the patient understood the content of the consultation. Spending adequate time with the patient is one of the most important predictors of patient satisfaction, which is linked to a greater adherence to therapy, return visit to the physician, and health improvements [29–33]. In our study, Bengali patients were more likely to receive sufficient time for medical consultation than that of the ethnic minority patients.

In terms of the patients’ perception of being satisfied with the visit to the physician, the Bengali patients had a higher likelihood of expressing their satisfaction toward the physician than that of their ethnic minority counterparts. In Bangladesh, there still exists a strong cultural and traditional myth that health and diseases exist more among ethnic minority groups. Therefore, strategies should be carried out to address this serious misconception by physicians adopting better patient-centered behavior.

In addition, the favorable communication behavior from the physician was significantly higher among the patients who had a higher level of education and who used the Internet. This suggests that they may have the self-efficacy to engage in communicative behaviors with their clinicians thus influencing the communication dynamics and PPCB ratings [28]. Patients’ age was also reported as an influencing factor for ethnic minority patients having better PPCB. It may be that older patients experience more severe diseases, which increases anxiety and increases the importance of communication style for the physician [34].

Patients’ having a pre-organized plan about the content of consultation before visiting the physician was also reported as an essential predictor for both ethnic groups reporting better patient-centered communication behavior. Usually, the person who has a plan about the content of the consultation with the physician can talk and participate more appropriately, making the interaction more effective. Indeed, a statistical relationship was found in this study (*p* < .001) between the pre-organized plan of the consultation and patients’ participation in decision making relating to the recommended treatment.

The present study reported that female physicians were more likely to communicate more effectively with Bengali patients than male physicians. Our findings are in line with previous studies [9, 35, 36], while Roter et al. [35] noted that female physicians prefer to use more patient-centered contact and promote more open and fair interaction than that of male physicians. Evidence has been found that female physicians tend to consider psychosocial issues through appropriate questioning and counseling, increased use of emotional and compassionate talk, and more constructive patient inputs. These elements were helpful for more effective patient-centered medical conversation [35].

It is worth to note that some variables were found to affect both Bengali patients and ethnic minority patients, such as a pre-organized plan about consultation before visit; while some variables only affected Bengali patients, such as level of education, internet use and gender of physician and some variables only affected ethnic minority patients, such as age, area of residence and appointment length. These differences may be explained by the discrepancy of socio-economic and cultural status between Bengali and ethnic minority patients, which is also worthy of a future qualitative study.

This study has some limitations. First, physicians’ patient-centered behaviors were depicted based on self-reported information from the patients, which is subject to reporting errors. Second, considering there is a long line of patients waiting for a physician to be seen in medical centers due to the improper ratio between the number of physicians and patients; thus, physicians are always busy dealing with a large number of patients. Therefore, physicians are reluctant to complete any questionnaire about research. Moreover, quantitative measures of physician-patient interaction were examined rather than the quality of communication.

## Conclusion

In conclusion, our research demonstrates that patients in concordant ethnic relationships report higher levels of physicians’ patient-centered communication. Bengali patients received more supportive communication behaviors (e.g., physicians’ cheerful greetings, affectionate behaviors, explaining the patients about a medical test, medication, and probable side effects, discussing the treatment options and involved the patients in decisions and spending adequate time) from the Bengali physicians than that of ethnic minority patients. Incorporating issues of cultural competency and social inequality into medical education and developing a culture-centered communication framework is an imperative aspect for reducing ethnic and socio-economic inequality. This will help ensure that all people can equally receive better health outcomes.

## Data Availability

All of the primary data has been included in the results. Additional materials with details may be obtained from the corresponding author.

## Abbreviations

ANOVA: Analysis of variance
BDT: Bangladeshi Taka
CHT: Chittagong Hill Tracts
PPCB: Physician-Patient Communication Behavior
UHC: Upazila Health Complex

## References

[1] Ashton CM, Haidet P, Paterniti DA, Collins TC, Gordon HS, O'Malley K (2003). Racial and ethnic disparities in the use of health services: bias, preferences, or poor communication?. J Gen Intern Med.

[2] Brown JB, Stewart M, Ryan BL. Outcomes of patient-provider interaction. In: The Routledge handbook of health communication. London: Routledge; 2003. p. 155–76.

[3] Gupta N, Carr NT (2008). Understanding the patient-physician interaction: potential for reducing health disparities. J Appl Soc Sci.

[4] Ong LM, De Haes JC, Hoos AM, Lammes FB (1995). Doctor-patient communication: a review of the literature. Soc Sci Med.

[5] Ahmed R (2012). Interpersonal health communication an ecological perspective. The handbook of global health communication.

[6] Cooper LA, Roter DL, Johnson RL, Ford DE, Steinwachs DM, Powe NR (2003). Patient-centered communication, ratings of care, and concordance of patient and physician race. Ann Intern Med.

[7] Cooper-Patrick L, Gallo JJ, Gonzales JJ, Vu HT, Powe NR, Nelson C, Ford DE (1999). Race, gender, and partnership in the patient-physician relationship. Jama.

[8] Doescher MP, Saver BG, Franks P, Fiscella K (2000). Racial and ethnic disparities in perceptions of physician style and trust. Arch Fam Med.

[9] Kaplan SH, Gandek B, Greenfield S, Rogers W, Ware JE. Patient and visit characteristics related to physicians’ participatory decision-making style: results from the medical outcomes study. Med Care. 1995:1176–87 https://www.jstor.org/stable/pdf/3766817.pdf. 10.1097/00005650-199512000-00002 7500658

[10] Saha S, Komaromy M, Koepsell TD, Bindman AB (1999). Patient-physician racial concordance and the perceived quality and use of health care. Arch Intern Med.

[11] Street RL, Thompson TL, Dorsey AM, Miller KI, Parrott R (2003). Communication in medical encounters: an ecological perspective. Handbook of health communication.

[12] Matusitz J, Spear J (2014). Effective doctor–patient communication: an updated examination. Soc Work Public Health.

[13] Smedley BD, Stith AY, Nelson AR (2003). Institute of Medicine, committee on understanding and eliminating racial and ethnic disparities in health care. Unequal treatment: confronting racial and ethnic disparities in healthcare.

[14] Harmsen H, Meevswesen L, Van Wieringen J, Bernsen R, Bruijnzeels M (2003). When cultures meet in general practice: intercultural differences between GPs and parents of child patients. Patient Educ Counsel.

[15] Berger JT (1998). Culture and ethnicity in clinical care. Arch Intern Med.

[16] Lloyd M, Bor R (1996). Communication skills for medicine.

[17] Barman DC, Neo MS (2014). Human rights report 2013 on indigenous peoples in Bangladesh.

[18] Uddin ME (2011). Cross-cultural social stress among Muslim, Hindu,Santal and Oraon communities in Rasulpur of Bangladesh. Int J Sociol Soc Pol.

[19] Uddin E (2015). Ethnic disparity in family socio-economic status in Bangladesh: implication for family welfare policy practice. Glob Soc Welf.

[20] Masud Ahmed S (2001). Differing health and health-seeking behaviour: ethnic minorities of the Chittagong Hill tracts, Bangladesh. Asia Pacific J Public Health.

[21] Saha PS (2015). Indigenous people sill in Back (Adibashira ekhono pichiye). The Prothom-alo.

[22] Tufnell DJ, Nutall K, Raistrick J, Jackson TL (1994). Use of translated written material to communicate with non-English speaking patients. Br Med J.

[23] Paternotte E, van Dulmen S, van der Lee N, Scherpbier AJ, Scheele F (2014). Factors influencing intercultural doctor-patient communication: a realist review. Patient Educ Couns.

[24] Ishikawa H, Takayama T, Yamazaki Y, Seki Y, Katsumata N (2002). Physician-patient communication and patient satisfaction in Japanese cancer consultations. Soc Sci Med.

[25] Andaleeb SS, Siddiqui N, Khandakar S (2007). Patient satisfaction with health services in Bangladesh. Health Policy Plan.

[26] Bangladesh Bureau of Statistics (BBS) (2020). Bangladesh statistics 2019.

[27] Ministry of Health and Family Welfare (MOHFW) (2018). Health bulletin 2017.

[28] Wachira J, Middlestadt S, Reece M, Peng CYJ, Braitstein P. Psychometric assessment of a physician-patient communication behaviors scale: the perspective of adult HIV patients in Kenya. AIDS Res Treatment. 2013;2013. 10.1155/2013/706191. 10.1155/2013/706191 PMC3582093 23476754

[29] Cegala DJ, Post DM (2009). The impact of patients’ participation on physicians’ patient centered communication. Patient Educ Couns.

[30] Grayson-Sneed KA, Dwamena FC, Smith S, Laird-Fick HS, Freilich L, Smith RC (2016). A questionnaire identifying four key components of patient satisfaction with physician communication. Patient Educ Couns.

[31] Levinson W, Lesser CS, Epstein RM (2010). Developing physician communication skills for patient-centered care. Health Aff.

[32] Marcum ZA, Sevick MA, Handler SM (2013). Medication nonadherence: a diagnosable and treatable medical condition. JAMA.

[33] Zolnierek KBH, DiMatteo MR (2009). Physician communication and patient adherence to treatment: a meta-analysis. Med Care.

[34] Buller MK, Buller DB. Physicians’ communication style and patient satisfaction. J Health Soc Behav. 1987:375–88. 10.2307/2136791. 3429807

[35] Roter DL, Hall JA, Aoki Y (2002). Physician gender effects in medical communication: a meta-analytic review. JAMA.

[36] Weisman CS, Teitelbaum MA (1989). Women and health care communication. Patient Educ Couns.

